# Functional Characteristics of Multipotent Mesenchymal Stromal Cells from Pituitary Adenomas

**DOI:** 10.1155/2016/7103720

**Published:** 2016-06-02

**Authors:** Kaspars Megnis, Ilona Mandrika, Ramona Petrovska, Janis Stukens, Vita Rovite, Inga Balcere, Laima Sabine Jansone, Raitis Peculis, Valdis Pirags, Janis Klovins

**Affiliations:** ^1^Latvian Biomedical Research and Study Centre, Ratsupites Street 1 k-1, Riga LV-1067, Latvia; ^2^Clinic of Neurosurgery, Pauls Stradins Clinical University Hospital, 13 Pilsonu Street, Riga LV-1002, Latvia; ^3^Centre of Endocrinology, Pauls Stradins Clinical University Hospital, 13 Pilsonu Street, Riga LV-1002, Latvia

## Abstract

Pituitary adenomas are one of the most common endocrine and intracranial neoplasms. Although they are theoretically monoclonal in origin, several studies have shown that they contain different multipotent cell types that are thought to play an important role in tumor initiation, maintenance, and recurrence after therapy. In the present study, we isolated and characterized cell populations from seven pituitary somatotroph, nonhormonal, and lactotroph adenomas. The obtained cells showed characteristics of multipotent mesenchymal stromal cells as observed by cell morphology, cell surface marker CD90, CD105, CD44, and vimentin expression, as well as differentiation to osteogenic and adipogenic lineages. They are capable of growth and passaging under standard laboratory cell culture conditions and do not manifest any hormonal cell characteristics. Multipotent mesenchymal stromal cells are present in pituitary adenomas regardless of their clinical manifestation and show no considerable expression of somatostatin 1–5 and dopamine 2 receptors. Most likely obtained cells are a part of tissue-supportive cells in pituitary adenoma microenvironment.

## 1. Introduction

Pituitary adenomas are typically slowly progressing benign intracranial endocrine tumors. They can be found in up to 14,4%–22,5% of population [1, 2]. Latest improvement in diagnostic techniques has led to an increasing incidence from 3,9 cases per 100 000 population in Sweden to 115,6 cases per 100 000 population in Iceland [3, 4].

Manifestation of clinically active adenomas can occur in three ways. Firstly, the adenoma can cause mass lesions by expanding in surrounding tissues, subsequently giving rise to headaches, visual field defects, and similar symptoms. Other two cases may lead to either pituitary hormone insufficiency or excess. Such hormonal alterations can lead to several syndromes, including acromegaly and Cushing's disease as well as several more common and less specific symptoms [5, 6]. Current medical therapies include transsphenoidal resection, pharmacotherapy with somatostatin or dopamine analogs, and irradiation but they have been proven to be insufficient in number of cases [7, 8].

Despite the suggested monoclonal origin of pituitary adenomas, several studies showed that more than one cell type can be found in pituitary adenoma [9, 10]. This can be explained by the fact that pituitary tumors may contain several tumor clones arising independently from expansion of individual cells [11]. On the other hand, there is a hypothesis that pituitary adenomas contain a subpopulation of tumor stem cells or other multipotent cells that drive their composition, growth, invasion, and resistance to therapy. They are suggested to be capable of sustaining themselves as well as differentiating into other cell types of the tumour [12].

It has been shown that pituitary adenomas contain self-renewing sphere-forming cell population that can give rise to stemness markers expressing spheres and it is considered as characteristic of cancer stem cells [13]. Although the concept of sphere formation in suspension culture as a proof of stemness has its drawbacks [14], expression of stem cell characteristic proteins, like nestin (NES), sex determining region Y box 2 (SOX2) or prominin 1 (PROM1, also known as CD133) [13, 15], should be mentioned. The origin of these cells is still under debate and can also be considered as a sign of differentiation. In normal pituitary, there are several nonhormonal cell types, like side population, colony-forming cells, or marginal cells, which manifest certain stem cell characteristics [16, 17]. In pituitary tumors, however, the picture is not that clear. Markers expressed by potential pituitary tumor stem cells overlap at some point with normal pituitary stem cell candidates but disparities are too big and information on this subject is too poor to draw the conclusions [12, 17]. Besides, several studies have shown clear expression of neural and glial cell markers in pituitary adenomas, which indicates possible involvement of surrounding tissue structures in pituitary tumorigenesis [18, 19].

In this study, we isolated cell populations from different types of pituitary adenomas and analysed them for expression of cell markers, differentiation potential, and pituitary hormone response.

## 2. Materials and Methods

### 2.1. Patients and Tissue Samples

All tissue samples and clinical information (Table 1) were obtained from planned resections at Centre of Endocrinology, Pauls Stradins Clinical University Hospital. Research was approved by Central Medical Ethics Committee of Latvia (permission 01-29.1/28). All patients had macroadenomas with extracellular extension. Two of them were clinically nonhormonal (patients did not have increased hormone level in their bloodstream), two were somatotrophic, and three were lactotrophic adenomas. Five of them were females, and two were males. Their age distribution varied from 26 to 74 years. For all patients this was their first pituitary adenoma. After resection, adenoma tissue samples were carefully separated from any nonadenoma tissues and divided into two parts. One part was submerged in RNAlater® Solution (Thermo Fisher Scientific, USA) for RNA extraction, and another part was immersed in Dulbecco's Modified Eagle Medium (DMEM) (Thermo Fisher Scientific, USA) for cell culture development.

### 2.2. Cell Culture Development

Tissue samples were disaggregated with scalpel, washed in DMEM with 1x Gibco® Antibiotic-Antimycotic solution (Thermo Fisher Scientific, USA), and fractured further by pipetting. Tumor cells were released from the tissues by enzymatic treatment with Accutase solution (Thermo Fisher Scientific, USA) for 20 min at 37°C on rotating platform in a humidified atmosphere maintained at 5% CO_2_. At the end of incubation, the cells were pelleted by centrifugation (5 min, 1600 rpm). Cells were grown in DMEM-F12 (Thermo Fisher Scientific, USA) with 1% L-glutamine (Thermo Fisher Scientific, USA), 10% Fetal Bovine Serum, ES Cell-Qualified (FBS) (Thermo Fisher Scientific, USA), 1% ITS Premix Universal Culture Supplement (Corning, USA), and 0,5% Primocin*™* (InvivoGen, USA) until reaching confluency. Cells above three passages on cell culture flasks were used for further experiments. All cell culture cultivations and incubations throughout the study were carried out at 37°C, 95% air, and 5% CO_2_.

### 2.3. Cell Proliferation Assay

Cell proliferation was tested by Cell Counting Kit-8 (CCK-8) (Sigma-Aldrich, USA), according to manufacturer's instructions. Cells (100 *μ*L) were seeded into 96-well tissue culture plate at the density of 10000 cells/well and grown overnight. CCK-8 solution (10 *μ*L) was added to the cells at the indicated times and incubated for 4 h at 37°C. The absorbance of each well was measured at 450 nm using a microplate reader (Victor3*™*, Perkin Elmer, USA). Data were expressed as a ratio of optical density (OD) at a specific time point over the initial OD on the fourth day. Wells without cells but containing medium were used as a blank value that was subtracted from all values. Each assay was performed in triplicate.

### 2.4. Testing of the Cell Hormonal Profile

Cell cultures from hormonal adenomas were incubated for 24 h in DMEM-F12 with 1% L-glutamine and 1% ITS Premix Universal Culture Supplement followed by 6 h incubation in medium with 10 nM concentrations of growth hormone releasing hormone (GHRH) for somatotrophic adenoma cultures or thyrotropin releasing hormone (TRH) (GenWay Biotech, USA) for lactotrophic adenoma cultures. Because of the short half-life of the hormones, medium was changed every 30 min. Cell cultures incubated with or without GHRH or TRH were immunocytochemically analysed using primary antibodies for growth hormone (GH) or prolactin (PRL) (Santa Cruz Biotechnology, USA) in order to test the presence of corresponding hormone.

### 2.5. RNA Extraction and Real Time PCR

RNA from tissues and cell cultures were extracted using mirVana*™* miRNA isolation kit (Thermo Fisher Scientific, USA) and treated with DNase using DNA-free*™* DNA removal kit (Thermo Fisher Scientific, USA). Total RNA (1 *μ*g) was reverse-transcribed into cDNA using random hexamer primer and RevertAid H Minus First Strand cDNA synthesis kit (Thermo Fisher Scientific, USA). Real time PCR analyses were conducted using VIIA*™*7 machinery and TaqMan® gene expression assays for genes: somatostatin receptor 1 (SSTR1) (Hs00265617_s1), somatostatin receptor 2 (SSTR2) (Hs00265624_s1), somatostatin receptor 3 (SSTR3) (Hs00265633_s1), somatostatin receptor 4 (SSTR4) (Hs01566620_s1), somatostatin receptor 5 (SSTR5) (Hs00265647_s1), dopamine receptor 2 (DRD2) (Hs00241436_m1), PROP paired-like homeobox 1 (PROP1) (Hs00395073_m1), paired-like homeodomain 1 (PITX1) (Hs00267528), ISL LIM homeobox 1 (ISL1) (Hs00158126_m1), POU class 1 homeobox 1 (POU1F1) (Hs00230821_m1), POU class 5 homeobox 1 (POU5F1) (Hs00999632_g1), sex determining region Y box 2 (SOX2) (Hs01053049_s1), and reference gene, tyrosine 3-monooxygenase/tryptophan 5-monooxygenase activation protein, zeta (YWHAZ) (assay ID Hs03044281_g1) (Thermo Fisher Scientific, USA). Each sample was run in triplicate and the mean value was normalized with respect to reference gene and used to calculate the relative amount of the targets. Each mean value was calculated using comparative (2^−ΔCt^) method.

### 2.6. Cell Characterization by Flow Cytometry

Cell surface antigen phenotype was performed on cells at passage 3–6. Cells were detached using trypsin 0.2% solution and counted. Cells were liquated at 1–5 × 10^5^ cells in tubes, pelleted, and resuspended in 100 *μ*L of phosphate buffered saline (PBS), pH 7.4, containing 0.5% bovine serum albumin (BSA). Then cells were stained with anti-human antibodies, CD90-fluorescein isothiocyanate (FITC) (eBioscience, USA), CD44-allophycocyanin (APC) (BioLegend, USA), CD45-V450 (BD Bioscience, USA), CD105- phycoerythrin (PE) (Santa Cruz Biotechnology, USA), CD34-PE (eBioscience, USA), and CD133-APC (Miltenyl Biotec, USA), at concentration of 1 *μ*g/mL at room temperature for 1 h. The respective isotype antibodies were used as negative controls. The cells were pelleted, washed twice with PBS-BSA buffer, and resuspended in 100 *μ*L buffer. Then, fluorescence-activated cell sorting (FACS) analysis was performed on BD Biosciences FACSAria flow cytometer (BD Biosciences, USA).

### 2.7. Immunofluorescent Staining

For immunofluorescent staining cells were washed with PBS and fixed with 4% paraformaldehyde for 15 min at room temperature. For permeabilization, 0.1% Triton X-100 was used. Cells were incubated for 1 h with primary antibodies for vimentin (VIM), nestin (NES), glial fibrillary acidic protein (GFAP), ataxin 1 (ATXN1), tubulin, and beta 3 class III (TUBB3) (Santa Cruz Biotechnology, USA) as well as A2B5 (binds to neural tissue such as brain, spinal cord, and dorsal root ganglia), oligodendrocyte lineage transcription factor 2 (OLIG2), and solute carrier family 1 member 3 (SLC1A3) (Thermo Fisher Scientific, USA). Nonimmune immunoglobulins of the same isotype as the primary antibodies were used as negative controls. Nuclei were stained with DAPI before mounting in ProLong Gold antifade reagent (Thermo Fisher Scientific, USA). Stained cells were visualized using a fluorescence microscope, Leica DM3000 (Leica Microsystems, Germany).

### 2.8. Osteogenic Differentiation

Osteogenic differentiation of the cells was induced using the osteogenic differentiation kit (PromoCell, Germany) according to the manufacturer's protocols. Cells were seeded into 24-well plate (50000 cells/well) and grown until they reach the confluency. The cells were induced for 21 days and the osteogenic medium was replaced every 2-3 days. Mineralization was assessed by staining the cells with 40 mM Alizarin Red S (pH 4.2) after fixation in 10% formalin.

### 2.9. Adipogenic Differentiation

Adipogenic differentiation of the cells was done by using adipogenic modulators as described [20] for 14 days. Lipid accumulation was assessed through triglyceride staining with Oil Red O dye (Sigma-Aldrich, USA). Cells were washed twice with PBS and fixed with 4% paraformaldehyde for 10 min at 37°C. Cells were rinsed with PBS and incubated with freshly diluted Oil Red O dye for 30 min at 37°C. Cells were washed again before visualization under a light microscope.

### 2.10. Neural and Glial Differentiation

Cells were seeded on Geltrex® substrate (Thermo Fisher Scientific, USA), in KnockOut*™* DMEM/F-12 (Thermo Fisher Scientific, USA), 1% L-glutamine, 0,5% Primocin, StemPro® NSC SFM (Thermo Fisher Scientific, USA), and 20 ng/mL EGF and bFGF. After two days medium was changed for specific cell type differentiation medium. For neural differentiation, medium was changed to Neurobasal® Medium (Thermo Fisher Scientific, USA) with 1% L-glutamine and 2% B-27® Serum-Free supplement. For astrocytic differentiation DMEM was used with 1% L-glutamine, 1% FBS, and 1% N-2 supplement (Thermo Fisher Scientific, USA). For oligodendrocytic differentiation, we used Neurobasal Medium with 1% L-glutamine, 2% B-27 Serum-Free supplement, and 30 ng/mL Triiodo-L-Thyronine (Sigma-Aldrich, USA). Medium was changed twice a week until first changes in cell morphology were observed.

## 3. Results

### 3.1. Cells Isolation and Proliferation

**Figure 1:**
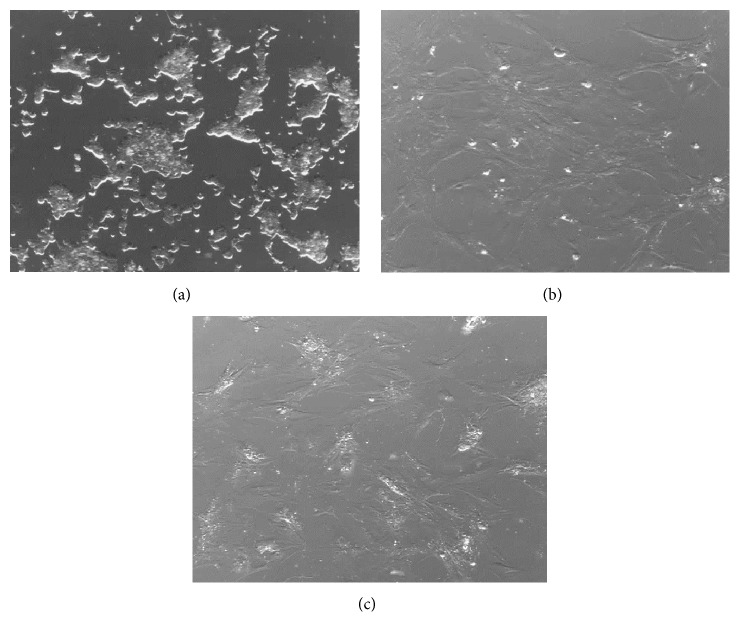
Representative example of pituitary adenoma cells observed under inverted microscopy after culture. (a) Initial cells after 2 days of culture; (b) culture of the cells after 14 days; (c) culture after 1 passage. Cell confluence and homogenous population were observed during cell passages. Magnification ×100. At the beginning of plating, cells displayed epithelial-like cell morphology which by time turned into fibroblast-like morphology.

Cell cultures were obtained from two nonhormonal, two somatotrophic, and three lactotrophic pituitary adenomas. Cells isolated from pituitary adenomas adhered to the surface of culture plastic plates within 24 h and showed epithelial-like cell morphology (Figure 1(a)). However, passaging of cells fraction resulted in growth of homogenous adherent cell population with fibroblast-like morphology (Figures 1(b)-1(c)). Moreover, cells obtained from different types of pituitary adenomas showed similar cell morphology.

**Figure 2:**
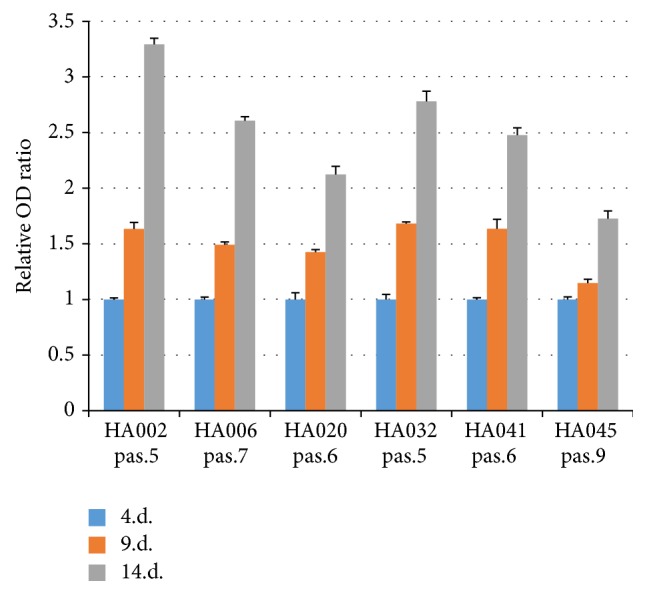
Proliferation rate of the cells isolated from pituitary adenoma samples. Cell cultures were evaluated at passages (pas.) 5–9. Values are mean ± SD from two experiments performed in triplicate.

Cell proliferation ability had no significant differences for cells obtained from different types of pituitary adenomas (Figure 2).

### 3.2. Cell Hormonal Profile Testing

Tumor tissue samples used in this study were immunohistochemically analysed for secretion of pituitary hormones according to hospital routine and researchers were provided with the data. Somatotrophic and lactotrophic adenomas were confirmed by GH and PRL production, respectively. In order to evaluate if obtained cells maintain hormone-secreting cells, cell cultures from somatotrophic and lactotrophic adenomas were stained for GH or PRL. We have not observed positive staining for GH or PRL in cell cultures (data not shown). To see if lack of hormonal content is caused by growth conditions rather than cell origin characteristics, we incubated cells in medium with hypothalamic hormones (GHRH and TRH) which naturally induces GH and PRL production in normal pituitary. After 6 h incubation with periodical culture medium change, cells still did not show any hormonal content when stained for GH or PRL (data not shown).

### 3.3. Expression of Pituitary Parenchymal Progenitor Markers

**Figure 3:**
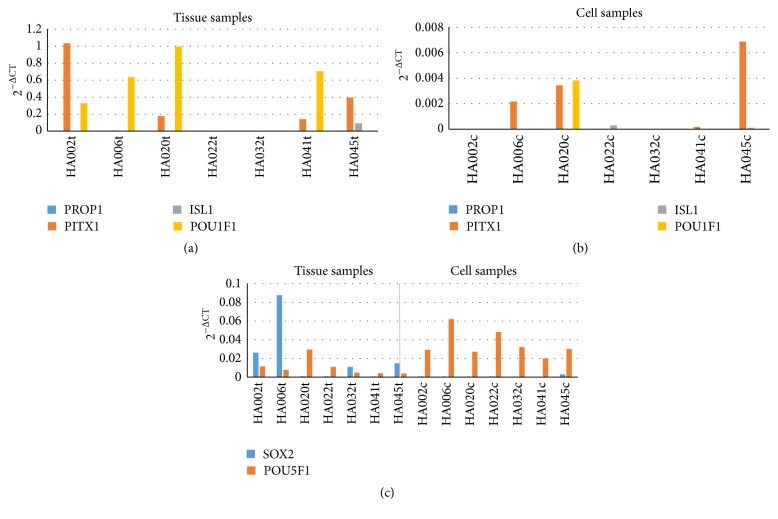
Expression of pituitary parenchymal progenitor cell markers in adenoma tissues and corresponding cell cultures. Values are calculated using comparative (2^−ΔCt^) method and mean Ct values from experiments performed in triplicate. Although adenoma tissue samples showed meaningful expression of markers from pituitary parenchymal cells and progenitors (a), their expression in cell cultures was substantially lower (b). PROP1, PROP paired-like homeobox; PITX1, paired-like homeodomain 1; ISL1, ISL LIM homeobox 1; POU1F1, POU class 1 homeobox 1; SOX2, SRY-box 2; POU5F1, POU class 5 homeobox 1.

To evaluate possible pituitary parenchymal cell involvement in adenoma formation, we analysed expression of several markers that are characteristic to pituitary parenchymal cells and their progenitors throughout organogenesis. For this study, we selected PROP1 (as embryonal marker), PITX1 (as oral ectoderm marker), ISL1 (as pouch progenitor marker), and POU1F1 (as PIT1 linage marker). The original tissue samples showed expression of PITX1, ISL1, and POU1F1, revealing presence of corresponding cell types (Figure 3(a)). However, the expression of these markers was much lower and inadequate in cell cultures (Figure 3(b)). It could indicate that gained cell cultures are not originated from Rathke's pouch, meaning that they are not pituitary parenchymal cells or their progenitors.

### 3.4. Analysis of Mesenchymal Stem Cell Markers

**Figure 4:**
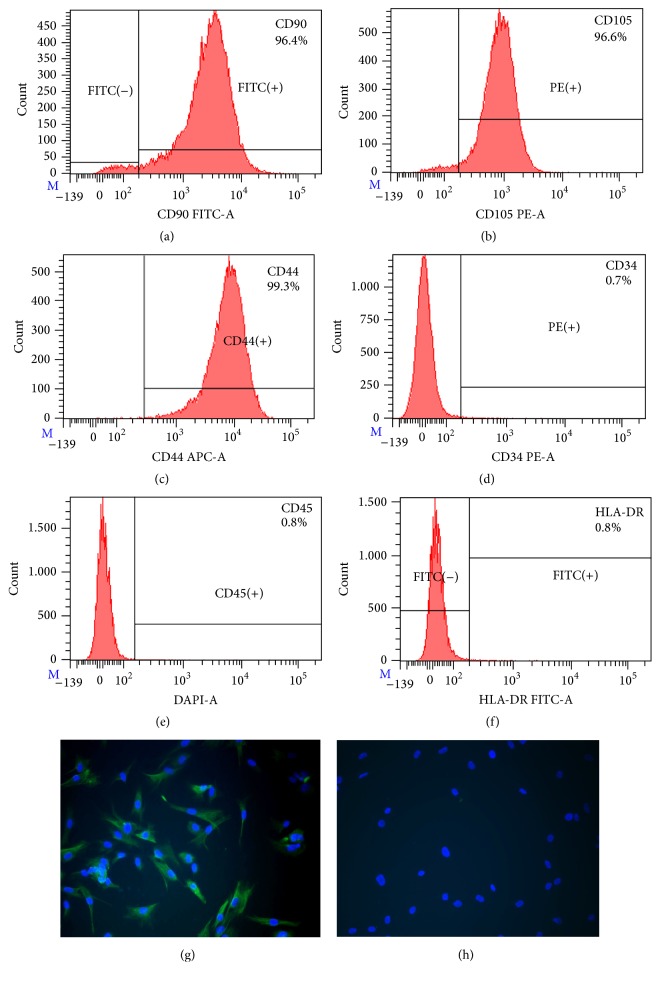
*In vitro* characterization of pituitary adenoma cell cultures for mesenchymal stromal cell markers. Representative FACS histograms of pituitary somatotroph adenoma cell culture (a–f). Cells were positive for CD90 (a), CD105 (b), and CD44 (c) and were negative for CD34 (d), CD45 (e), and HLA-DR (f). Representative immunostaining of VIM (green) (g) and negative control (h). Nuclei were stained with DAPI (blue). 200x magnification. CD90, Thy-1 cell surface antigen; CD105, endoglin; CD44, CD44 molecule (Indian blood group); CD34, CD34 molecule; CD45, protein tyrosine phosphatase, receptor type, C; HLA-DR, major histocompatibility complex, class II, DR.

The expression of mesenchymal stem cell markers in pituitary adenoma cell cultures was analysed by flow cytometry and immunofluorescent staining. Analysis of cell marker expression on obtained pituitary adenoma cells revealed similar phenotype in all types of cells between passages 3 and 6. All samples strongly expressed mesenchymal stem cell markers such as CD90 (Thy-1 cell surface antigen), CD105 (endoglin), and CD44 (hyaluronic acid receptor) (Figures 4(a)–4(c)). However, the proportion of CD90^+^ cell population was variable between obtained cell cultures, ranging from 63.4% to 99.6%. All samples were negative for CD34 (a marker of bone marrow cells), CD45 (hematopoietic stem cell marker), and human leukocyte antigen-DR (HLA-DR) (Figures 4(d)–4(f)). Furthermore, the cells showed expression of cytoskeletal protein, VIM (Figure 4(g)), which is ubiquitously expressed in normal mesenchymal cells and is known as a marker for epithelial-mesenchymal transition. Expression of the stem cell markers SOX2 and POU5F1 (Oct3/4) was examined by real time PCR. Although SOX2 and POU5F1 expression in adenoma tissue and cell cultures was very low, it was detectable in all samples (Figure 3(b)).

CD133 has been used as a marker for stem cells in normal and cancer tissues. It has been shown that in pituitary adenomas CD133 expression correlated with tumor cell invasiveness. FACS analysis of CD133 in obtained pituitary cell cultures showed no noticeable expression of this cell marker (data not shown).

### 3.5. Osteogenic and Adipogenic Differentiation

**Figure 5:**
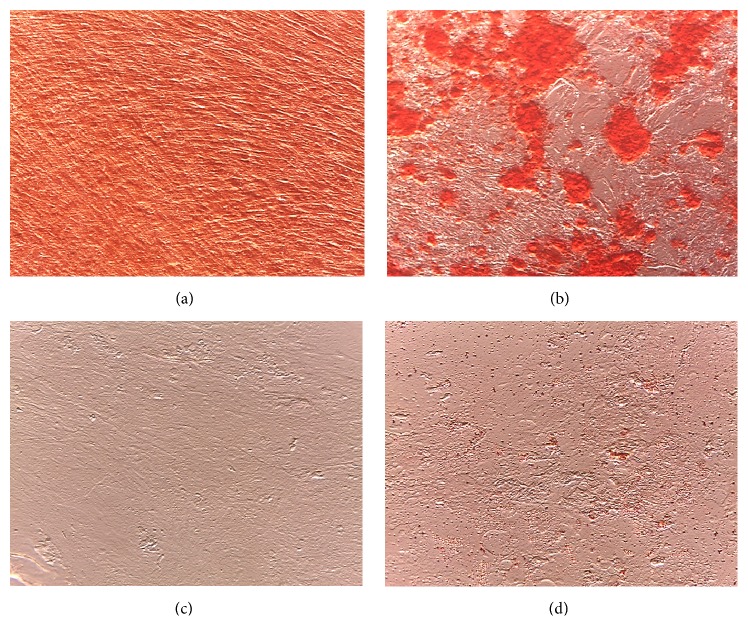
Pituitary adenoma cell cultures exhibited competence to differentiate into osteogenic and adipogenic lineages upon specific induction. Osteogenic differentiation was confirmed by Alizarin Red S staining of calcium inclusions (control (a), differentiated (b)). Magnification ×200. Adipogenic differentiation was confirmed by Oil Red O staining of lipid droplets (control (c), differentiated (d)). Magnification ×400 (c-d).

To study multilineage capacity of obtained cell cultures, cells were differentiated toward the osteogenic and adipogenic lineages using lineage-specific induction factors. To confirm their osteogenic capacity, cells were treated with osteogenic differentiation medium for 21 days, and the formation of calcified extracellular matrix was assessed using Alizarin Red S stain. Consistent with osteogenesis, plenty of red regions, indicative of a calcified extracellular matrix, were observed in differentiated cells but not in control cells (Figures 5(a)-5(b)). For adipogenic differentiation, cells were treated with adipogenic differentiation medium for 14 days, and accumulation of lipid droplets was assessed using Oil Red O stain. Staining of lipid droplets was observed only in differentiated cells (Figures 5(c)-5(d)).

### 3.6. Analysis of Proteins Associated with Neural Lineages

**Figure 6:**
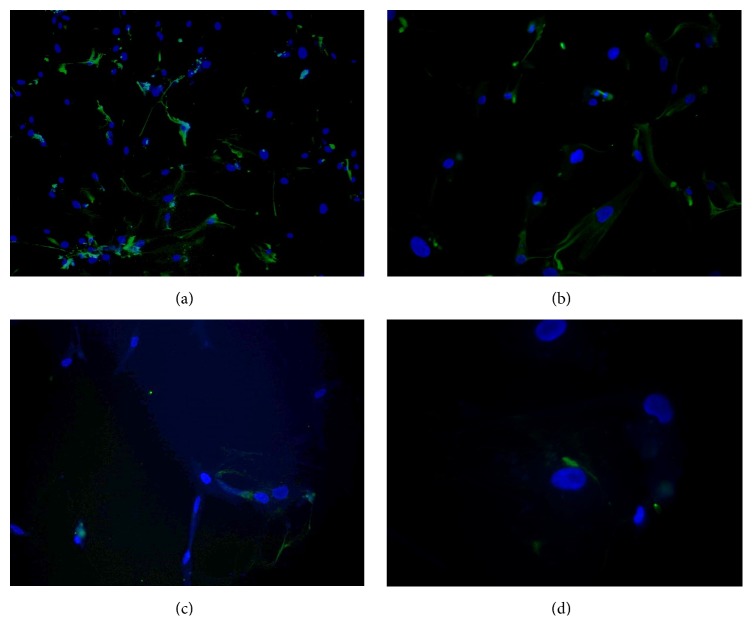
Immunohistochemical analysis of proteins associated with neural lineages in pituitary adenoma cell cultures. (a) HA002, NES (green), 100x magnification; (b) HA006, NES (green), 200x magnification; (c) HA006, A2B5 (green), 200x magnification; (d) HA032, ATXN1 (green), 200x magnification. Nuclei were stained with DAPI (blue). All analysed samples showed great NES expression, mild A2B5 and GFAP staining, and, in several cells, very weak ATXN1 expression. NES, nestin; A2B5 binds to neural tissue such as brain, spinal cord, and dorsal root ganglia; GFAP, glial fibrillary acidic protein; ATXN1, ataxin 1.

To analyse possible involvement of neural and glial precursor cells in development of pituitary adenomas, cells were stained for NES, A2B5, GFAP, and ATXN1 (also known as SCA1, known to be expressed in pituitary side population cells). All analysed samples showed high NES expression, mild A2B5 and GFAP staining, and, in a few cells, very weak ATXN1 expression (Figure 6). Although NES can be expressed in various cell types, in adulthood its expression is mainly limited to neural precursors. Therefore, its expression combined with A2B5 and GFAP may indicate presence of neural precursors or partly differentiated cells from neural or glial cell lineages.

### 3.7. Neural and Glial Differentiation

**Figure 7:**
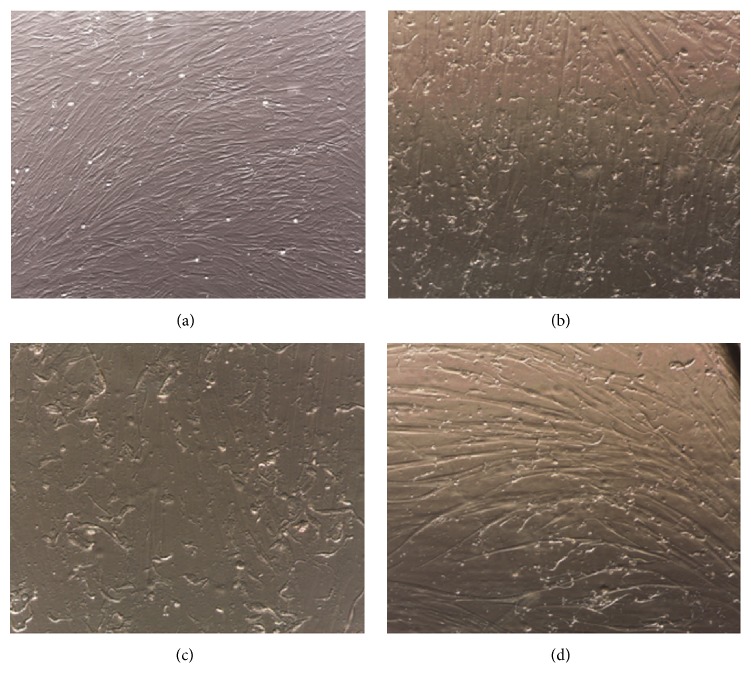
Cell morphological changes after differentiation into neural and glial cells. HA002 before differentiation (a) and after astrocyte differentiation (b), 100x magnification; HA006 after oligodendrocyte differentiation (c), 200x magnification; HA032 after neural differentiation (d), 200x magnification. Morphological cell changes were observed during neural and glial differentiation; cells developed multiple small irregular dendrite-like protrusions.

Positive NES and A2B5 staining encouraged us to test the ability of these cells to differentiate into neurons, atrocities, and oligodendrocytes. Cell differentiations were carried out until first changes in cell morphology were observed (Figure 7). Originally, mesenchymal-like cells (Figure 7(a)) developed multiple small irregular dendrite-like protrusions (Figures 7(b)–7(d)). Although morphological changes were promising and equal for all samples and differentiation techniques, immunocytochemical analysis showed only barely noticeable expression of TUBB3 (as neuronal marker), SLC1A3 (as astrocytes marker), and OLIG2 (as oligodendrocytes marker) (data not shown). Thus, we cannot present the convincing evidence of neural and glial differentiation for these cells.

### 3.8. Somatostatin and Dopamine Receptor Expression

**Figure 8:**
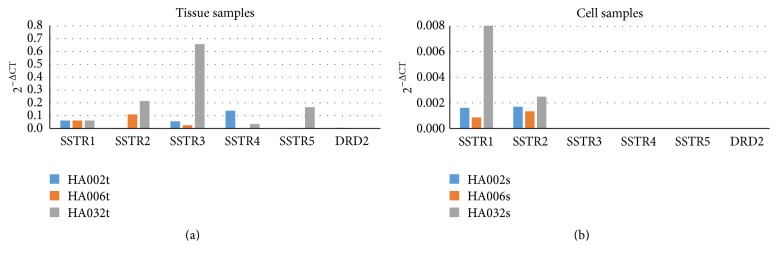
Somatostatin and dopamine receptor expression in tissue samples (a) and corresponding cell cultures (b). Values are calculated using comparative (2^−ΔCt^) method and mean Ct values from experiments performed in triplicate. Although adenoma tissue samples showed meaningful expression of somatostatin and dopamine receptors (a), their expression in cell cultures was not substantial (b). SSTR1–5, somatostatin receptors 1 to 5; DRD2, dopamine receptor D2.

In order to analyse potential of obtained cells to respond to somatostatin and dopamine analogues commonly used in medicamental treatment of pituitary adenomas, we have measured expression of their target receptors in pituitary adenoma tissues and cell cultures. Although tissue samples showed different expression levels of SSTR1–5 and DRD2 receptors, their expression in cell cultures was much lower and detectable in only few certain cases (Figure 8). From these results, we can estimate that somatostatin and dopamine analogues' ability to affect these pituitary adenoma cells could be very limited.

## 4. Discussion

Pituitary adenomas are usually slow progressing benign tumors. Although they are rarely malignant and do not possess high mortality, their [21] impact on other organ systems has aroused interest among many researchers [21, 22]. Not long ago, it was believed that pituitary adenomas consist only of mutated pituitary hormonal cells. However, growing evidence suggests that their cellular composition is more complex and richer with different cell types [17, 23]. In the present study, we obtained cell cultures from seven different pituitary adenomas and analysed their characteristics, potential, and possible origin.

All cell cultures, regardless of pituitary adenoma characteristics, displayed fibroblast-like cell morphology and showed no hormonal response to hypothalamic hormones, which naturally induce production of pituitary hormones. We however did not observe any difference in their growth characteristics. Thus, in our experiments, cells obtained from somatotroph and lactotroph adenomas were not able to induce their origin specific hormone production. It may indicate that either obtained cell cultures have lost their hormonal-producing potential during performed procedures* in vitro*, or are not differentiated hormonal cells, or these cells are originated from pituitary stroma. We cannot exclude the fact that experimental conditions were not adequate to resemble the exact pituitary environment and inability to achieve the hormone production could be attributed to the lack of specific additional factors. On the other hand, the ability of gained cells to extensively proliferate regardless of their original tumor type suggests that obtained cell cultures may consist of stromal cells, which are strong enough to sustain* in vitro* cultivation and are commonly present in pituitary adenomas. In order to avoid the possible overgrowth of the cultures with fibroblasts at the beginning of the study we examined number of tissues in parallel using DMEM medium containing L-valine versus D-valine, which has been reported to prevent fibroblast growth due to the absence of D-amino acid oxidase in these cells [24]. We however did not observe any difference in their growth characteristics.

To see if our obtained cell cultures were originated from Rathke's pouch, we tested them for presence of markers that are normally expressed throughout different time points of pituitary organogenesis [25]. As it was expected, tissue samples showed different expression levels of these markers, but their expression in cell cultures was barely noticeable, indicating presence of pituitary parenchymal cells and their progenitors in adenoma tissues but not in cell cultures.

Analysis of mesenchymal stem cell markers (CD44, CD90, CD105, and VIM) expression as well as differentiation ability of these cells into osteoblasts, adipocytes, and partly neural and glial cells indicated that our obtained cell cultures are most probably mesenchymal stem-like cells that for some reasons are located in pituitary adenomas. Analysis of cell proliferation and self-renewal indicated that although cells manifest certain multipotency, their stemness is questionable. For this reason, it would be more suitable to use the term multipotent mesenchymal stromal cells instead of mesenchymal stem cells [26]. Although multipotent mesenchymal stromal cells are normally found in almost all tissues and are relevant for proper functioning of most of the organ systems, they have been recorded to have essential role in tumour formation and progression [27–29].

Obtained cell cultures showed limited capacity of differentiation into neural and glial cells. These results may indicate that gained cell cultures are able to prelude neural and glial differentiation but not complete it, as it is typical to multipotent mesenchymal stromal cells [30, 31]. Positive staining for NES, GFAP, and A2B5 in untreated cell samples made us conclude that these cells already possess the features of these cell types because of partial differentiation most probably into adenoma tissue. These results partly relate to Johnson and colleagues' results, where they found cells in pituitary adenoma tissues, which were morphologically in neural transdifferentiation state, as well as cells with positive phosphoneurofilament, Class III b-tubulin and Neu-N immunoreactivity [18].

Unlike tissue samples, cell cultures presented very low expression of somatostatin and dopamine receptors and only in certain cases. It could possibly indicate that these multipotent mesenchymal stromal cells may not respond to somatostatin and dopamine analogues, widely used in medicamental therapy of pituitary adenomas. It would partly explain common recurrence of adenomas after therapy.

By now, several studies have detected different stem-like cell subpopulations in pituitary adenomas. Yunoue with colleagues identified cell population in pituitary adenomas that coexpressed CD133 and CD34 [15]. In our obtained cell cultures, both these markers were negative. As mentioned before, they did not show noteworthy expression of pituitary parenchymal markers as well, and even cultures from hormonal adenomas did not respond to hypothalamic hormone treatment. It may indicate that our obtained cell culture is not the only one multipotent cell culture in pituitary adenomas. Studies have shown that CD133 expression is essential for stem cell tumorigenesis [32]. Its absence in obtained cell cultures could possibly indicate that these cells are not the main CSCs population that ensures tumorigenesis but they could be some kind of tumour supportive cells. Future studies will show if there is any credibility in this theory.

Differences in obtained cell cultures between studies could be explained by usage of different cell culture isolation techniques. Tumor sphere formation methodology with EGF, FGF, and B-27 supplement seems to be usable for gaining pituitary adenoma stem/progenitor cells that might be capable of tumor formation and differentiation into hormonal cells [19, 33]. Our applied methodology is usable for gaining multipotent mesenchymal stromal cells with no hormonal differentiation capacity [34, 35].

## 5. Conclusion

In conclusion, our studies show multipotent mesenchymal stromal cells' presence in pituitary adenomas. Although it has been observed before [34], we indicate that their presence has no relation with clinical manifestation of adenoma or patients' age. They showed no response to hypothalamic hormones that normally induce production of pituitary hormones and expression of somatostatin and dopamine receptors in these cells is very limited. Further studies are necessary to evaluate their involvement in formation and progression of pituitary adenomas.

## Figures and Tables

**Table 1:** Pituitary adenoma samples characterization.

Samples ID	Hormonal activity	Patient age	Patient sex
HA002	Nonhormonal	53	Female
HA006	Lactotroph	74	Female
HA020	Somatotroph	26	Female
HA022	Lactotroph	31	Male
HA032	Somatotroph	53	Female
HA041	Lactotroph	50	Male
HA045	Nonhormonal	55	Female
